# Comparison of chemical profiles, antioxidation, inhibition of skin extracellular matrix degradation, and anti-tyrosinase activity between mycelium and fruiting body of *Cordyceps militaris* and *Isaria tenuipes*

**DOI:** 10.1080/13880209.2021.2025255

**Published:** 2022-01-22

**Authors:** Adchara Prommaban, Suwannee Sriyab, Pachabadee Marsup, Waranya Neimkhum, Jakkapan Sirithunyalug, Songyot Anuchapreeda, Chaiwat To-anun, Wantida Chaiyana

**Affiliations:** a Department of Pharmaceutical Sciences, Faculty of Pharmacy, Chiang Mai University, Chiang Mai, Thailand; b Faculty of Pharmacy, Chiang Mai University, Chiang Mai, Thailand; c Department of Pharmaceutical Technology, Faculty of Pharmaceutical Sciences, Huachiew Chalermprakiet University, Samutprakarn, Thailand; d Research Center of Pharmaceutical Nanotechnology, Chiang Mai University, Chiang Mai, Thailand; e Division of Clinical Microscopy, Department of Medical Technology, Faculty of Associated Medical Sciences, Chiang Mai University, Chiang Mai, Thailand; f Department of Entomology and Plant Pathology, Faculty of Agriculture, Chiang Mai University, Chiang Mai, Thailand; g Innovation Center for Holistic Health, Nutraceuticals, and Cosmeceuticals, Faculty of Pharmacy, Chiang Mai University, Chiang Mai, Thailand

**Keywords:** Cordycepin, adenosine, anti-ageing, anti-skin wrinkle

## Abstract

**Context:**

*Cordyceps militaris* and *Isaria tenuipes* (Cordycipitaceae) are high-value fungi that are used for health-promoting food supplements. Since laboratory cultivation has begun for these fungi, increased output has been achieved.

**Objective:**

This study compared the chemical profiles, antioxidant, anti-tyrosinase, and skin extracellular matrix degradation inhibition between mycelium and fruiting body of *C. militaris* and *I. tenuipes*.

**Materials and methods:**

The antioxidative potential of 10% v/v aqueous infused extract from each fungus was separately investigated using 2,2-azinobis(3-ethylbenzo-thiazoline-6-sulphonic acid) (ABTS), 1,1-diphenyl-2-picrylhydrazyl (DPPH), ferric reducing antioxidant ability, and ferric thiocyanate methods. The inhibition against MMP-1, elastase, and hyaluronidase were determined to reveal their anti-wrinkle potential. Anti-tyrosinase activities were determined.

**Results:**

*C. militaris* and *I. tenuipes* extracts were found to contain a wide range of bioactive compounds, including phenolics, flavonoids, and adenosine. A correlation was discovered between the chemical compositions and their biological activities. The extract from *I. tenuipes* fruiting body (IF) was highlighted as an extraordinary elastase inhibitor (IC_50_ = 0.006 ± 0.004 mg/mL), hyaluronidase inhibitor (IC_50_: 30.3 ± 3.2 mg/mL), and antioxidant via radical scavenging (ABTS IC_50_: 0.22 ± 0.02 mg/mL; DPPH IC_50_: 0.05 ± 0.02 mg/mL), thereby reducing ability (EC_1_: 95.3 ± 4.8 mM FeSO_4_/g extract) and lipid peroxidation prevention (IC_50_: 0.40 ± 0.11 mg/mL). IF had a three-times higher EC_1_ value than ascorbic acid and significantly higher elastase inhibition than epigallocatechin gallate.

**Discussion and conclusions:**

IF is proposed as a powerful natural extract with antioxidant and anti-wrinkle properties; therefore, it is suggested for further use in pharmaceutical, cosmeceutical, and nutraceutical industries.

## Introduction

Entomopathogenic fungi, a type of fungal pathogen that infects a wide range of insect species, are divided into five categories: Chytridiomycota (Chytrids), the Zygomycota (conjugated fungi), the Ascomycota (sac fungi), the Basidiomycota (club fungi), and the recently described phylum Glomeromycota (Litwin et al. 2020). Among *Cordycipitaceae*, *Cordyceps militaris* (L.) Fr. Link (Dong-Chung-Ha-Cho) and *Isaria tenuipes* Peck (Snowflake Dong-Chung-Ha-Cho) are the authentic fungal supplies for food ingredients, herbal products, and dietary supplements in various Asian countries (Moon et al. 2018). *C. militaris* has been reported to grow in many geographic locations and can be artificially cultivated in the laboratory (Nxumalo et al. 2020). *C. militaris* is one of the well-known *Cordyceps* that have been used in traditional Chinese medicine for over a century as health-promoting supplements (Ng and Wang 2005). *C. militaris* has been shown to have a variety of biological functions, including antioxidant, immunomodulatory, hypolipidemic, and antitumor properties (Ng and Wang 2005; Marsup et al. 2020).

Apart from *C. militaris*, *I. tenuipes* is also an important edible and medicinal fungal species due to its various beneficial pharmacological activities. Nowadays, *I. tenuipes* can also be cultivated in controlled laboratories and is available as a health food and traditional medicine for anti-ageing, antioxidation, antibacterial use, antidepression, antitumor, lowering blood glucose and blood fat, etc. (Seo et al. 2013; Zhang et al. 2019). In addition, *I. tenuipes* has been used as folk medicine for strengthening the immune system and regaining energy in Japan, China, and South Korea, whereas it has been used as a tonic for recovery from tuberculosis and for speedy recovery after childbirth in the Himalayan region (Chhetri et al. 2020).

**Figure 1.**
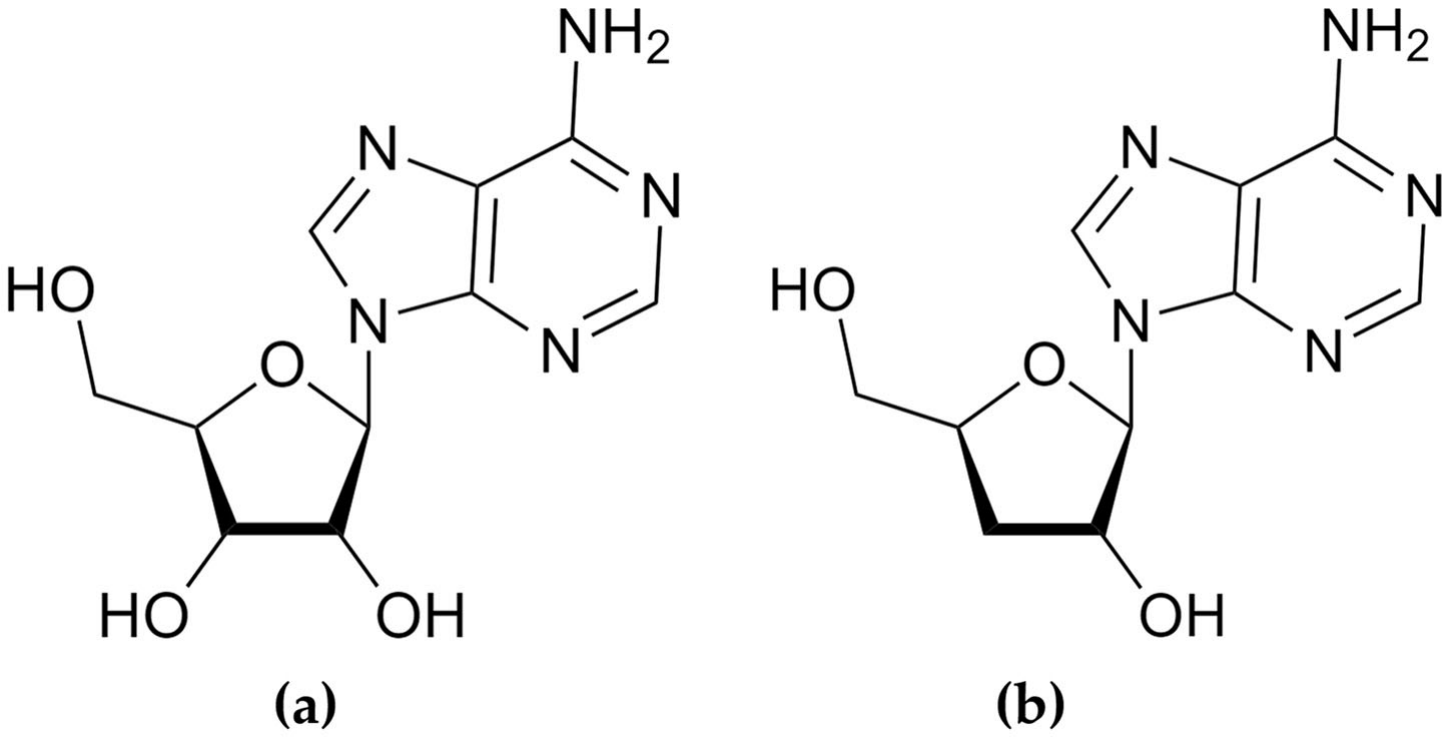
Chemical structure of adenosine (a) and cordycepin (b).

Cordycepin, produced by secondary metabolism in *C. militaris* and *I. tenuipes*, has a structure similar to adenosine (Figure 1) (Suparmin et al. 2017; Litwin et al. 2020). With age, the extracellular levels of adenosine decline (Tescarollo et al. 2020). The increase of adenosine content in human peripheral blood mononuclear cells after nicotinamide riboside supplementation has resulted in anti-ageing effects and health benefits (Jacobson and Reitman 2020). Since the chemical structure of cordycepin is similar to that of adenosine, it might inhibit adenosine-dependent processes (Litwin et al. 2020). Therefore, both *C. militaris* and *I. tenuipes* could have anti-ageing potential.

According to the chemical compositions and various biological activities of *C. militaris* and *I. tenuipes*, and accompanied with the ability to be cultivated in the laboratory, it is interesting to apply these fungi as active ingredients for anti-ageing. However, there are very few studies investigating the anti-skin ageing and anti-wrinkle activities of *C. militaris* and *I. tenuipes* extracts, this is the first study to investigate the anti-skin wrinkle activities of *C. militaris* and *I. tenuipes* extracts.

## Materials and methods

### Chemical materials

Analytical grade cordycepin (purity ≥98.0%), adenosine (purity ≥99.0%), l-ascorbic acid (purity ≥99.0%), epigallocatechin gallate (EGCG; purity ≥95.0%), oleanolic acid (purity ≥97.0%), sodium chloride (NaCl), sodium phosphate (Na_3_PO_4_), sodium dihydrogen phosphate (NaH_2_PO_4_), disodium phosphate (Na_2_HPO_4_), sodium carbonate (Na_2_CO_3_), tricine, tris base, elastase from porcine pancreas lyophilised powder (E–E.C.3.4.21.36), *N*-succinyl-Ala-Ala-Ala-*p*-nitroanilide (AAAPVN), metalloproteinase-1 (MMP-1) from *Clostridium histolyticum* (Weinberg and Seguin 1916) (ChC–EC.3.4.23.3), *N*-[3-(2-furyl)acryloyl]-Leu-Gly-Pro-Ala (FALGPA; purity ≥99.0%), hyaluronidase from bovine testes (E.C.3.2.1.3.5), hyaluronic acid, bovine serum albumin (BSA), and sodium lauryl sulphate (SLS) were purchased from Sigma–Aldrich (St. Louis, MO, USA). Analytical grade ethanol, hexane, ethyl acetate, acetic acid, and dimethyl sulfoxide (DMSO) were purchased from Labscan (Dublin, Ireland). HPLC-grade methanol was purchased from Labscan (Dublin, Ireland).

### Fungi materials

*C. militaris* and *I. tenuipes*, obtained fresh from the Mushroom Research and Development Centre (MRDC), Chiang Mai, Thailand, were identified by Prof. Dr. Chaiwat To-anun, an expert in fungal biodiversity at the Department of Entomology and Plant Pathology, Faculty of Agriculture, Chiang Mai University, Chiang Mai, Thailand. The fruiting bodies of *C. militaris* and *I. tenuipes* were separated from their mycelium and dried in a hot air oven (Memmert GmbH & Co. KG, Schwabach, Germany) set at 40 °C overnight until dry. The dried fungi were ground into fine powder using a Moulinex DB81 blender (Moulinex, Paris, France) and kept in a well-closed container until further use.

### Extraction of *C. militaris* and *I. tenuipes*

*C. militaris* and *I. tenuipes* were extracted by infusion in boiling DI water. Briefly, 5 mL of boiling DI water was added to 1 g of dried fungi material and mixed for 5 min using a vortex mixer Genie 2 (Scientific Industries, NY, USA). The resulting mixture was then centrifuged at 6,000 rpm for 10 min at ambient temperature using a Thermo Fisher Scientific centrifuge (Sorvall ST16R, Waltham, MA, USA). Subsequently, the supernatant was collected and filtered through a 0.45 μm nylon syringe filter (Whatman Puradisc 25, USA). Four extracts were obtained, including *C. militaris* fruiting body extract (CF), *C. militaris* mycelium extract (CM), *I. tenuipes* fruiting body extract (IF), and *I. tenuipes* mycelium extract (IM). The colour of each extract was determined using the free online program from https://html-colour-codes.info/colors-from-image/. The extract was kept in the refrigerator until the further experiment.

### Determination of chemical compositions of *C. militaris* and *I. tenuipes* extracts

#### Total phenolics content determination by the Folin–Ciocalteu method

The Folin–Ciocalteu method was used to assess the total phenolics content of *C. militaris* and *I. tenuipes* extracts, as previously described by Chaiyana et al. (2017). Gallic acid was used as a standard compound and the total phenolic contents were expressed as milligrams per gram of gallic acid equivalents (GAE). Three independent experiments, repeated in triplicate, were performed.

#### Total flavonoid content determination by the aluminium chloride method

The aluminium chloride colorimetric method was used to assess the total flavonoid content of *C. militaris* and *I. tenuipes* extracts (Chaiyana et al. 2020). Quercetin was used as a standard compound and the total flavonoid content was expressed as quercetin equivalent (QE). Three independent experiments, repeated in triplicate, were performed.

#### Cordycepin and adenosine content determination by high performance liquid chromatography (HPLC)

HPLC (Hewlett Packard, Palo Alto, CA, USA) with C-18 reverse phase column (Zorbac Eclipse XDB-C18, 4.6150 mm, 0.5 m) and guard column (Zorbac Eclipse XDB-C18, 4.650 mm, 0.5 m) was used to evaluate the cordycepin and adenosine content of *C. militaris* and *I. tenuipes* extracts. To elute the sample, a mobile phase consisting of 5% methanol and 95% of DI water containing 0.05% w/v formic acid was used at a flow rate of 1 mL/min. The sample with an injection volume of 20 μL was detected by the UV detector set at a wavelength of 260 nm. The cordycepin and adenosine standard curves were plotted using a concentration of standard compounds varying from 1 to 150 µg/mL. The tests were carried out twice. Cordycepin content was calculated using the equation: *x* = (*y* + 2.6553)/6.3672 (*R*^2^ = 0.9998), where *x* is the cordycepin content and *y* is the area under the curve (AUC) of cordycepin peak detected around 7.4 min in the HPLC chromatogram. On the other hand, adenosine content was calculated using the equation: *x* = (*y − 6.309*)/5.9824 (*R*^2^ = 0.9976), where x is the adenosine content and y is the area under the curve (AUC) of adenosine peak detected around 5.9 min in the HPLC chromatogram.

### Antioxidant activities determination

#### 2,2′-Azinobis (3-ethylbenzothiazoline-6-sulphonic acid) (ABTS) assay

ABTS assay was used to determine the ABTS^•+^ radical scavenging activity of *C. militaris* and *I. tenuipes* sample solutions (Chaiyana et al. 2019). The ABTS^•+^ scavenging activity was calculated using the equation: % ABTS^•+^ scavenging activity = [1 – (*A*/*B*)] × 100, where *A* is the UV absorbance of the mixture containing sample solution and *B* is the UV absorbance of the sample solution-free mixture. IC_50_ values were then calculated from the graph plotted inhibition percentage against the concentration of sample solution using the Graphpad/Prism program version 2.01 (Graphpad Software Inc., La Jolla, CA, USA). Ascorbic acid was used as a positive control. Three independent experiments, repeated in triplicate, were performed.

#### 1,1-Diphenyl-2-picrylhydrazyl radical scavenging (DPPH) assay

DPPH assay was used to determine the DPPH^•^ radical scavenging activity of *C. militaris* and *I. tenuipes* sample solutions (Chaiyana et al. 2019). The DPPH^•^ scavenging activity was calculated using the equation: % DPPH^•^ scavenging activity = [1 – (*A*/*B*)] × 100, where *A* is the UV absorbance of the mixture containing sample solution and *B* is the UV absorbance of the sample solution-free mixture. IC_50_ values were then calculated from the graph plotted inhibition percentage against the concentration of sample solution using the Graphpad/Prism program version 2.01 (Graphpad Software Inc., La Jolla, CA, USA). Ascorbic acid was used as a positive control. Three independent experiments, repeated in triplicate, were performed.

#### Ferric reducing antioxidant power (FRAP) assay

FRAP assay was used to determine the ferric reducing antioxidant power of *C. militaris* and *I. tenuipes* sample solutions (Chaiyana et al. 2017). FeSO_4_ was used as a standard compound and the ferric reducing antioxidant power was expressed as equivalent concentration (EC_1_) representing the concentration of sample solution with a reducing effect equivalent to 1 mM FeSO_4_. Three independent experiments, repeated in triplicate, were performed.

#### Ferric thiocyanate (FTC) assay

FTC assay was used to determine the inhibitory activity on lipid peroxidation of *C. militaris* and *I. tenuipes* sample solutions (Osawa and Namiki 1981). The lipid peroxidation inhibition was calculated using the equation: % lipid peroxidation inhibition = [1 – (*A*/*B*)] × 100, where *A* is the UV absorbance of the mixture containing sample solution and *B* is the UV absorbance of the sample solution-free mixture. IC_50_ values were then calculated from the graph plotted inhibition percentage against the concentration of sample solution using the Graphpad/Prism program version 2.01 (Graphpad Software Inc., La Jolla, CA, USA). Trolox was used as a positive control. Three independent experiments, repeated in triplicate, were performed.

### Anti-skin ageing activities determination

#### Determination of matrix metalloproteinase-1 (MMP-1) inhibition

The spectrophotometric method was used to investigate the inhibitory activity against MMP-1 of *C. militaris* and *I. tenuipes* sample solutions (Thring et al. 2009; Chaiyana et al. 2019). The MMP-1 inhibition was calculated using the equation: % MMP-1 inhibition = [1 – (*A*/*B*)] × 100, where *A* is the UV absorbance of the mixture containing sample solution and *B* is the UV absorbance of the sample solution-free mixture. IC_50_ values were then calculated from the graph plotted inhibition percentage against the concentration of sample solution using the Graphpad/Prism program version 2.01 (Graphpad Software Inc., La Jolla, CA, USA). Oleanolic acid was used as a positive control. Three independent experiments, repeated in triplicate, were performed.

#### Elastase enzyme inhibition

The spectrophotometric method was used to investigate the inhibitory activity against elastase of *C. militaris* and *I. tenuipes* sample solutions (Thring et al. 2009; Chaiyana et al. 2019). The elastase inhibition was calculated using the following equation: % Elastase inhibition = [1 – (*A*/*B*)] × 100, where *A* is the UV absorbance of the mixture containing sample solution and *B* is the UV absorbance of the sample solution-free mixture. IC_50_ values were then calculated from the graph plotted inhibition percentage against the concentration of sample solution using the Graphpad/Prism program version 2.01 (Graphpad Software Inc., La Jolla, CA, USA). Epigallocatechin gallate (EGCG) was used as a positive control. Three independent experiments, repeated in triplicate, were performed.

#### Hyaluronidase inhibition

The spectrophotometric method was used to investigate the inhibitory activity against hyaluronidase of *C. militaris* and *I. tenuipes* sample solutions (Nema et al. 2011). The hyaluronidase inhibition was calculated using the equation: % Hyaluronidase inhibition = [1 – (*A*/*B*)] × 100, where *A* is the UV absorbance of the mixture containing sample solution and *B* is the UV absorbance of the sample solution-free mixture. Oleanolic acid was used as a positive control. Three independent experiments, repeated in triplicate, were performed.

### Antityrosinasse activities determination

The spectrophotometric method was used to investigate the inhibitory activity against tyrosinase activity of *C. militaris* and *I. tenuipes* sample solutions (Saeio et al. 2011). The tyrosinase inhibition was calculated using the equation: % Tyrosinase inhibition = [1 – (*A*/*B*)] × 100, where *A* is the UV absorbance of the mixture containing sample solution and *B* is the UV absorbance of the sample solution-free mixture. Kojic acid was used as a positive control. Three independent experiments, repeated in triplicate, were performed.

### Statistical analysis

The results from each triplicate experiment were expressed as mean ± standard deviation (S.D.) and their comparisons were analysed by using analysis of variance (ANOVA), followed by a Tukey *post hoc* test. Pearson correlation analysis was applied to determine the relationship between the chemical constituents of the fungi and their antioxidant activities. A *p*-value of less than 0.05 indicates statistical significance. The rule of thumb for interpreting the size of a correlation coefficient used was: correlation 0.90 to 1.00 (−0.90 to −1.00) as very high positive (negative) correlation; 0.70 to 0.90 (−0.70 to −0.90) as high positive (negative) correlation; 0.50 to 0.70 (−0.50 to −0.70) as moderate positive (negative) correlation; 0.30 to 0.50 (−0.30 to −0.50) as low positive (negative) correlation; and 0.00 to 0.30 (−0.00 to −0.30) as negligible correlation (Mishra et al. 2018).

## Results and discussion

### *C. Militaris* and *I. tenuipes* extracts

**Figure 2.**
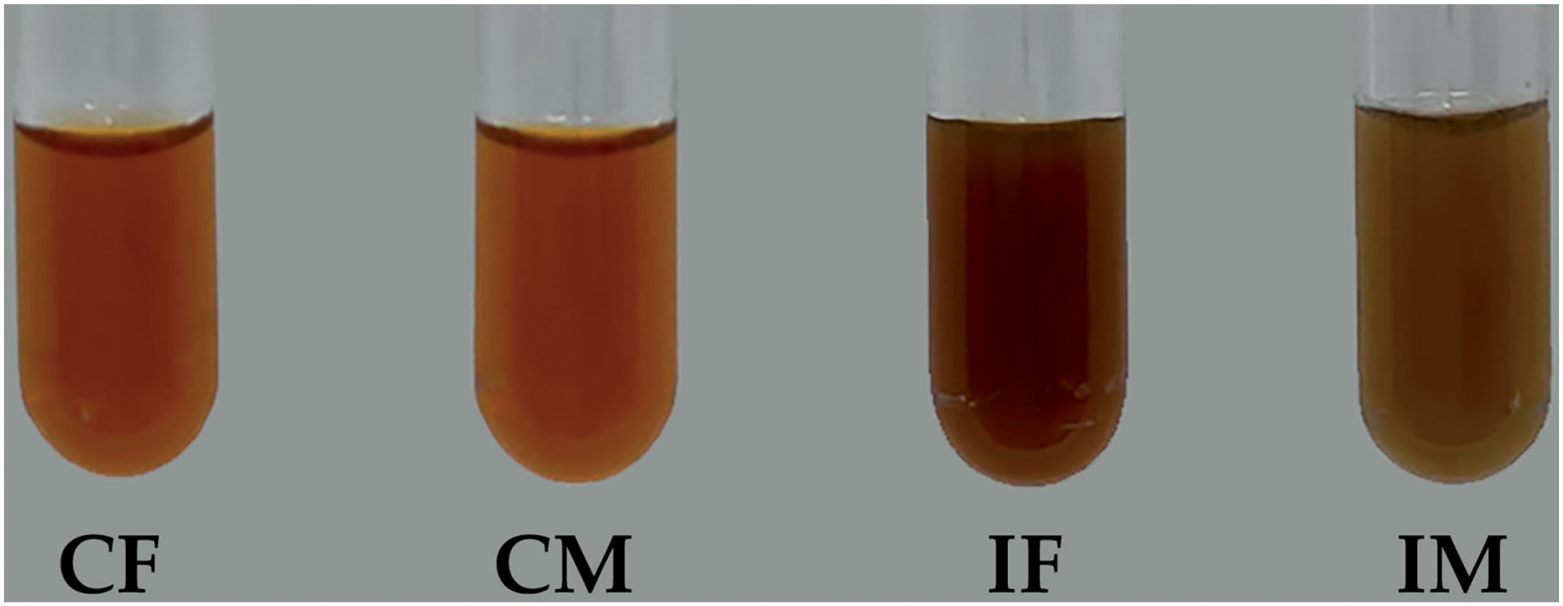
The external appearance of *C. militaris* fruiting body extract (CF), *C. militaris* mycelium extract (CM), *I. tenuipes* fruiting body extract (IF), and *I. tenuipes* mycelium extract (IM).

Both fruiting body and mycelium of *C. militaris* and *I. tenuipes* could be extracted using boiling DI water. The external appearance of each extract is shown in Figure 2. The orange-brown colour of the extracts from the fruiting body and mycelium of *C. militaris* is similar. This colour was known to be generated by photoinduced carotenogenesis, which resulted in the production of a variety of carotenoid pigments, including lutein, zeaxanthin, cordyxanthins, and cordycepene (Shrestha et al. 2006; Dong et al. 2013). These pigments have been identified as primary compounds responsible for the orange-brown colour of both the fruiting body and the mycelium of *C. militaris* (Shrestha et al. 2006). On the other hand, the extract from *I. tenuipes* had a darker colour. Moreover, the extract from the fruiting body of *I. tenuipes* was noticeably darker than the mycelium part as shown in Table 1; the reasons might be due to the production of pink, red, and reddish-brown pigments from *Isaria* spp. (Velmurugan et al. 2010). The results are in obvious accordance with the shades of water-soluble pigment generated by *Isaria* spp. previously reported by Velmurugan et al. (2010).

**Table 1.** Hex colour code, RGB colour code model, and CMYK colour code model of extracts from *C. militaris* and *I. tenuipes*.

Extracts	Hex colour code	RGB colour code model			CMYK colour code model			
		Red	Green	Blue	Cyan	Magenta	Yellow	Black
CF	#682900	40.78%	16.08%	0%	0%	61%	100%	59%
CM	#692800	41.18%	15.69%	0%	0%	62%	100%	59%
IF	#350802	20.78%	3.14%	0.78%	0%	85%	96%	79%
IM	#492600	28.63%	14.9%	0%	0%	48%	100%	71%

CF: *C. militaris* fruiting body extract; CM: *C. militaris* mycelium extract; IF: *I. tenuipes* fruiting body extract; IM: *I. tenuipes* mycelium extract.

### Chemical compositions of *C. militaris* and *I. tenuipes* extracts

**Figure 3.**
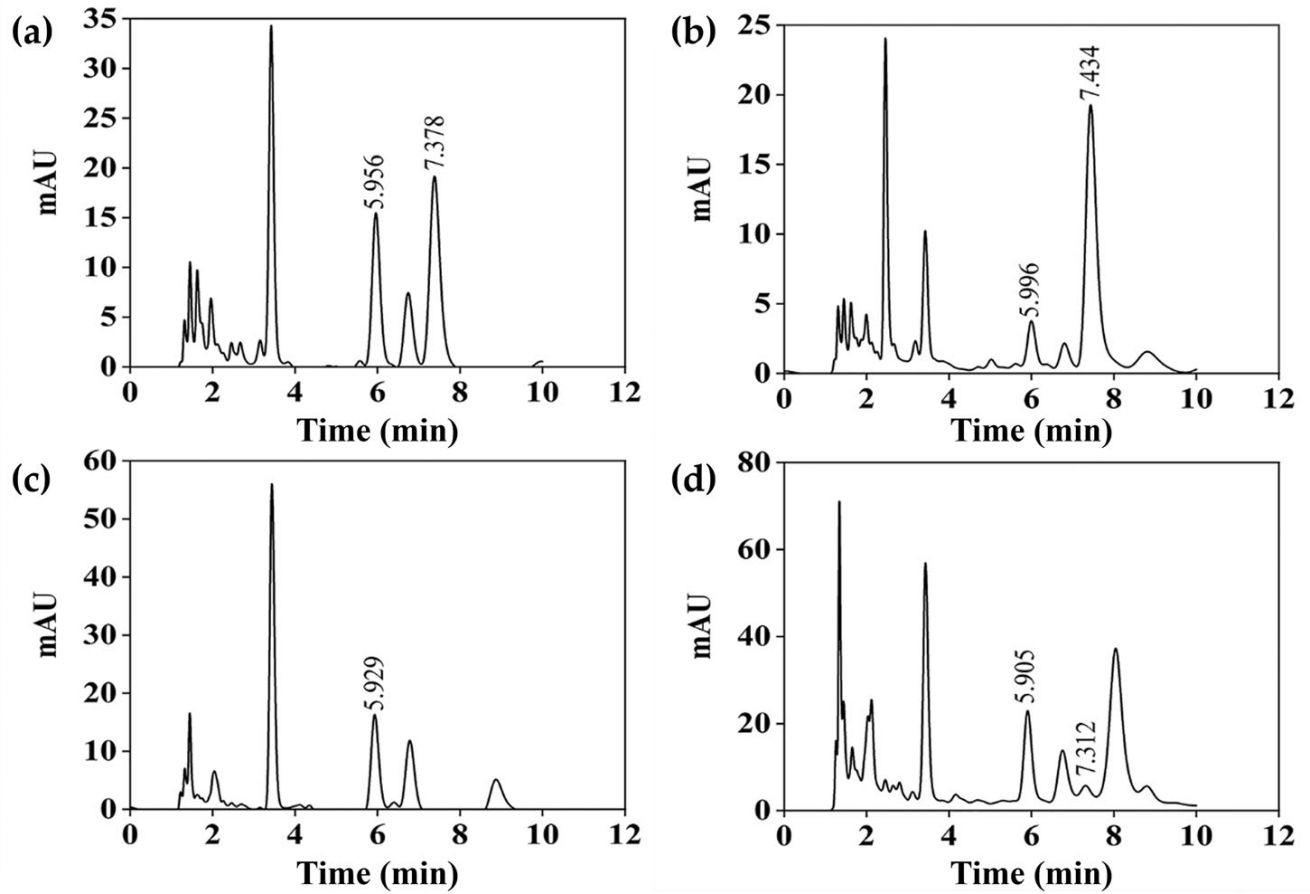
HPLC chromatograms of *C. militaris* fruiting body extract (CF) (a), *C. militaris* mycelium extract (CM) (b), *I. tenuipes* fruiting body extract (IF) (c), *I. tenuipes* mycelium extract (IM) (d).

HPLC chromatograms as shown in Figure 3 reveals the presence of cordycepin (peak detected around 7.4 min) and adenosine (peak detected around 5.9 min) in *C. militaris* and *I. tenuipes* extracts. The chemical compositions of the extracts are shown in Table 2. The fruiting body of both *C. militaris* and *I. tenuipes* contained significantly higher phenolic compounds than their mycelium part (*p* < 0.05). In contrast, the total flavonoids and cordycepin content were found to be identical in the fruiting body and mycelium. Adenosine content was also similar in the fruiting body and mycelium of *I. tenuipes*, but significantly higher in the *C. militaris* fruiting body compared to its mycelium part.

**Table 2.** Total phenolics, total flavonoids, cordycepin, and adenosine content of *C. militaris* and *I. tenuipes* extracts.

Extracts	TPC (mg GAE/g extract)	TFC (mg QE/g extract)	Cordycepin (mg/g extract)	Adenosine (mg/g extract)
CF	8.27 ± 0.04^a^	12.2 ± 1.5^a,b^	3.02 ± 0.11^a^	1.99 ± 0.01^a^
CM	2.46 ± 0.07^c^	11.0 ± 0.2^b^	3.00 ± .004^a^	0.44 ± 0.02^b^
IF	6.27 ± 0.01^b^	18.5 ± 1.3^a^	0.00 ± 0.00^c^	1.95 ± 0.00^a^
IM	0.82 ± 0.01^d^	13.5 ± 1.9^a,b^	0.08 ± 0.01^b^	1.99 ± 0.01^a^

CF: *C. militaris* fruiting body extract; CM: *C. militaris* mycelium extract; IF: *I. tenuipes* fruiting body extract; IM: *I. tenuipes* mycelium extract; TPC: total phenolics content; GAE: gallic acid equivalent; TFC: total flavonoids content; QE: quercetin equivalent. The letter a, b, c, and d denotes significantly different among samples at *p* < 0.05 after One-way ANOVA followed by Tukey's post hoc test.

Despite the fact that no prior studies had compared the phenolic content of the fruiting body and mycelium parts of *C. militaris* and *I. tenuipes*, the results were consistent with the study of *Ganoderma lucidum* (FR.) Karst (Ganodermataceae) (Chandrasekaran et al. 1978). Heleno et al. (2012) observed that the fruiting body of *G. lucidum* contained the highest phenolics content of 28.64 mg GAE/g extract, which was significantly higher than that found in mycelium grown in any culture medium, including solid Melin–Norkans medium (14.22 ± 0.29 mg GAE/g extract), liquid Melin–Norkans medium (6.03 ± 0.98 mg GAE/g extract), and Potato Dextrose Agar medium (15.19 ± 0.59 mg GAE/g extract). Mishra et al. (2018) also reported that the fruiting body of *G. lucidum* had the highest phenolics content of 4.13 μg GAE/g extract compared to its mycelium part. In contrast, the mycelium part of *Coprinus comatus* (O.F. Müll.) Pers. (Agaricaceae) and *Coprinellus truncorum* (Scop.) Redhead, Vilgalys & Monclavo (Psathyrellaceae) contained significantly higher phenolic content compared to the fruiting body (Tešanović et al. 2017). Therefore, the different distribution of phenolic compounds in each part of the fungus depended on the varying bioactive compound synthesis mechanism of each fungus species (Park et al. 2013).

In contrast to the phenolics content, flavonoid content was not significantly different in the fruiting body and the mycelium part of both *C. militaris* and *I. tenuipes* (Table 2). Findings were consistent with those of Carvajal et al. (2012), who observed no significant differences in flavonoid content in the fruiting body (1.8 ± 0.16 mg catechin/g extract), young mycelia (2.1 ± 0.20 mg catechin/g extract), and old mycelia (2.3 ± 0.20 mg catechin/g extract) of *Agaricus brasiliensis* Wasser. (Agaricaceae) On the other hand, a significant flavonoid content has been reported in several edible mushrooms (Barros et al. 2007). Flavonoids were found to be significantly more abundant than phenolics in all extracts in the present study (*p* < 0.05). The findings were consistent with those of Zhu et al. (2013), who found that the flavonoid content of both fresh and dry *C. militaris* fruiting bodies (25.74 ± 1.14 and 16.34 ± 1.23 mg rutin/g extract) was significantly higher than that of phenolics (1.93 ± 0.08 and 1.85 ± 0.07 mg gallic acid/g extract).

Similar to the flavonoid content, cordycepin was present in a comparable amount in both the fruiting body and the mycelium part of *C. militaris* (Table 2). However, the cordycepin distribution in the fruiting body or mycelium of *C. militaris* was variable. In some cases, cordycepin was higher in the fruiting body (2.654 ± 0.02 mg/g) compared to the mycelium (0.9040 ± 0.02 mg/g) (Huang et al. 2009), but in another case, mycelial biomass of *C. militaris* had higher cordycepin content (0.182 ± 0.08% w/w) than that in the fruiting body (0.110 ± 0.03% w/w) (Chan et al. 2015). On the other hand, no cordycepin was detected in *I. tenuipes* in the present study. This was consistent with the previous report noting that cordycepin was not detected in *I. tenuipes* but was considered a bioactive component of *Cordyceps* spp. and some *Isaria* spp., such as *Isaria cicadae* Miquel (Zhang et al. 2019).

Adenosine, a crucial parameter for *Cordyceps* quality control, was found to be higher in the fruiting body than the mycelium part (Table 2). The results were in accordance with the study of Huang et al. (2009) reported that the fruiting body of *C. militaris* contained higher adenosine (2.45 ± 0.03 mg/g) than the mycelium (1.592 ± 0.03 mg/g) (Zhu et al. 2013). On the other hand, comparable adenosine was detected in the fruiting body and mycelium of *I. tenuipes* (Table 2). Although there have been few studies comparing the adenosine content of *I. tenuipes* fruiting body and mycelium, the fruiting body has been previously reported to contain adenosine (Kang et al. 2010; Ji et al. 2011; Pham et al. 2020).

### Antioxidant activities of *C. militaris* and *I. tenuipes* extracts

**Figure 4.**
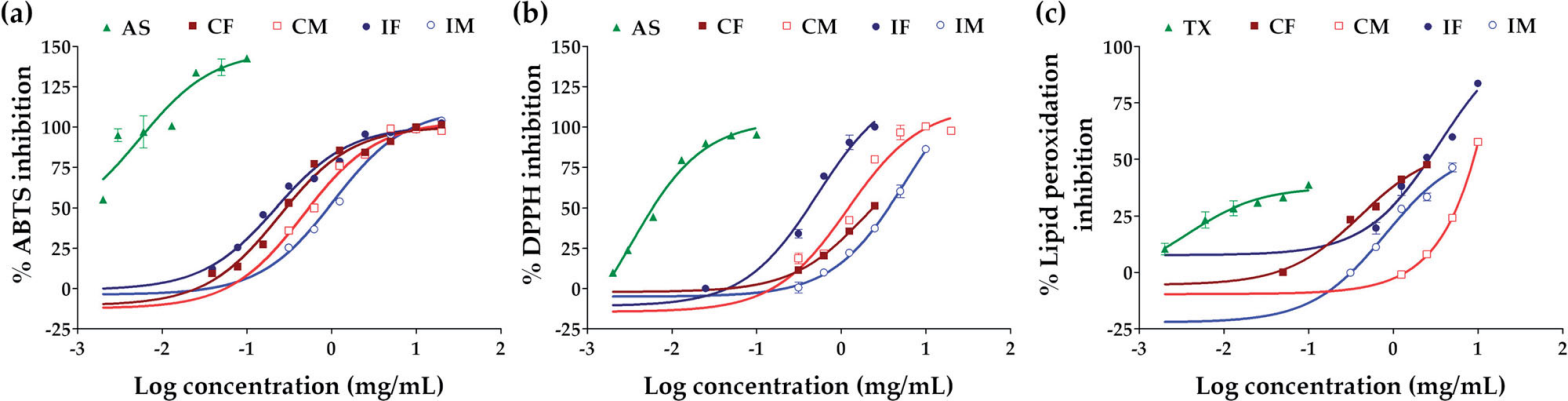
Dose-response curve on the inhibition of ABTS^•+^ (a), DPPH^•^ (b), and lipid peroxidation (c) of ascorbic acid (AS), Trolox (TX), *C. militaris* fruiting body extract (CF), *C. militaris* mycelium extract (CM), *I. tenuipes* fruiting body extract (IF), and *I. tenuipes* mycelium extract (IM).

The extracts of *C. militaris* and *I. tenuipes* inhibited ABTS^•+^, DPPH^•^, and lipid peroxidation in a dose-dependent manner (Figure 4) and the dose-response curve was used to quantify the IC_50_ value as presented in Table 3. IF was an extract with extraordinary antioxidant activities via radical scavenging activity, reducing ability, and prevention of lipid peroxidation. The most distinguished antioxidant activity of IF was via reducing ability since the EC_1_ value of IF was about three times higher than that of ascorbic acid, a well-known natural antioxidant.

**Table 3.** Antioxidant activities of *C. militaris* and *I. tenuipes* extracts.

Extracts	EC_1_ (mM FeSO_4_/g extract)	IC_50_ (mg/mL)		
		ABTS^•+^ inhibition	DPPH inhibition	Lipid peroxidation inhibition
AS	36.2 ± 1.6^b^	0.004 ± 0.000^a^	0.003 ± 0.000^a^	N.D.
TX	N.D.	N.D.	N.D.	0.04 ± 0.02^a^
CF	14.3 ± 0.5^c^	0.24 ± 0.02^b^	1.64 ± 0.35^b^	0.38 ± 0.08^b^
CM	29.4 ± 2.3^b^	0.46 ± 0.01^c^	1.20 ± 0.18^a,b^	5.53 ± 1.83^c^
IF	95.3 ± 4.8^a^	0.22 ± 0.02^b^	0.05 ± 0.02^a^	0.40 ± 0.11^b^
IM	13.9 ± 2.7^c^	1.05 ± 0.05^d^	5.18 ± 1.05^c^	0.49 ± 0.08^b^

IC_50_: a concentration that exhibited 50% inhibitory activity; EC_1_: equivalent concentration; AS: ascorbic acid; TX: Trolox; CF: *C. militaris* fruiting body extract; CM: *C. militaris* mycelium extract; IF: *I. tenuipes* fruiting body extract; IM: *I. tenuipes* mycelium extract; N.D.: not determined. The letter a, b, c, and d denote significant differences among samples at *p* < 0.05.

In contrast to *I. tenuipes*, the differences in antioxidant activity between the fruiting body and the mycelium of *C. militaris* were observed in the present study. This is well related to previous research reporting that the fruiting body and mycelium of *C. militaris* had their own role and worked in different ways. The mycelium of *C. militaris* exhibited stronger chelating ability, reducing power, and total antioxidant capacity than the fruiting body, which have been shown to have higher DPPH^•^ radical scavenging activity (Dong et al. 2014).

The relationship between the chemical constituents and antioxidant activities of the extracts as shown in Table 4 indicated adenosine content as a factor strongly affecting the lipid peroxidation inhibitory activity (*p* < 0.0006). The higher adenosine content resulted in a lower IC_50_ value, indicating more potent inhibitory activity since a lower concentration was needed for 50% inhibitory activity. The results were related to those of a previous study, which found that adenosine could interact with adenosine receptors (A1 and A3AR), causing the antioxidant enzymes to be activated and resulting in a reduction in lipid peroxidation in cells (Maggirwar et al. 1994; Ford et al. 1997). Since an increase in lipid peroxidation end products, such as lipid alkoxyl (LO^•^) and lipid peroxyl (LOO^•^) radicals, is the most widely cited evidence for free radical involvement in human disease, the extracts which possessed strong lipid peroxidation inhibition would be beneficial for human’s health (Ayala et al. 2014).

**Table 4.** The relationship between the chemical constituents and antioxidant activities of *C. militaris* and *I. tenuipes* extracts.

Chemical constituents	Pearson correlation coefficient; *r* (*p*-value)			
	EC_1_	ABTS^•+^ inhibition	DPPH^•^ inhibition	Lipid peroxidation inhibition
TPC	0.2980	−0.8613	−0.6558	−0.4063
	(0.7020)	(0.1387)	(0.3442)	(0.5937)
TFC	0.8842 (0.1158)	−0.2489 (0.7511)	−0.3339 (0.6661)	−0.5687 (0.4313)
Cordycepin	−0.5052	−0.4093	−0.2934	0.5637
	(0.4948)	(0.5907)	(0.7066)	(0.4363)
Adenosine	0.1276	0.0686	0.2638	−0.9994***
	(0.8724)	(0.9314)	(0.7362)	(0.0006)

Asterisks (***) denote significant strong correlations at *p* < 0.001.

On the other hand, the findings from this study indicated phenolic compounds as the major chemical constituents responsible for the radical scavenging activities of the extracts since the total phenolic content possessed a high correlation with the ABTS^•+^ inhibition (*r* = −0.8613) and moderate correlation with the DPPH^•^ inhibition (*r* = *−0.6558*) (Table 4). The negative correlations revealed that higher phenolics content was associated with a lower IC_50_ value, suggesting more potent radical scavenging activity because a lower concentration was required for the 50% inhibition. The findings were in good agreement with previous research indicating that phenolic compounds are essential chemical constituents that facilitate free radical scavenging through their hydroxyl groups (Soobrattee et al. 2005).

Furthermore, flavonoids were found to be responsible for the reducing abilities with a moderate correlation (*r* = 0.8842) in the present study (Table 4); related to a previous report that found flavonoids to be capable of reducing Fe^3+^ to Fe^2+^ (Mira et al. 2002). Since the presence of high-reducing-capacity reductants is highly correlated with potential antioxidant activity (Olugbami et al. 2014), IF with the highest EC_1_ value would be a potent antioxidant.

### Anti-wrinkle activities of *C. militaris* and *I. tenuipes* extracts

**Figure 5.**
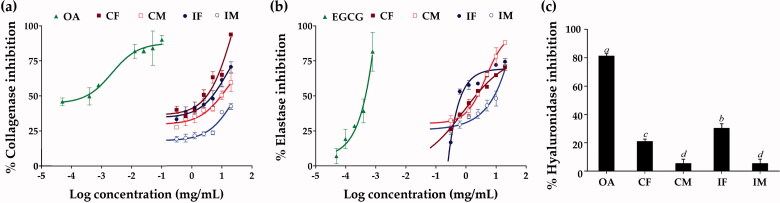
Dose-response curve on the inhibition of collagenase (a) and elastase (b), and hyaluronidase inhibition (c) f oleanolic acid (OA), epigallocatechin gallate (EGCG), *C. militaris* fruiting body extract (CF), *C. militaris* mycelium extract (CM), *I. tenuipes* fruiting body extract (IF), and *I. tenuipes* mycelium extract (IM). The letter a, b, c, and d denote significant differences among samples at *p* < 0.05.

Skin ageing naturally occurs from a decline in the extracellular matrix (ECM) in the dermis, including collagen fibres, elastin fibres, and hyaluronan by matrix metalloproteinase-1 (MMP-1), elastase, and hyaluronidase, respectively (Maity et al. 2011; Kim et al. 2019). Therefore, the inhibition of these enzymes can slow ECM deterioration and delay the appearance of wrinkles on the skin. Figure 5 depicts the inhibitory activity of *C. militaris* and *I. tenuipes* extracts on MMP-1, elastase, and hyaluronidase. The extracts of *C. militaris* and *I. tenuipes* inhibited MMP-1 and elastase in a dose-dependent manner and their IC_50_ value is shown in Table 5. CF was found to be the most effective inhibitor of MMP-1, while IF was found to be the most effective inhibitor of elastase (*p* < 0.05). Although the bioassay results indicated CF as the most effective inhibitor to MMP-1 in all extracts, its inhibitory activity was not very potent when compared with the positive control (oleanolic acid). The MMP-1 inhibitory effect of oleanolic acid was about 1,000 times more potent than CF. On the other hand, the elastase inhibitory effect of IF was close to the positive control (EGCG). Only a small amount of IF was required to inhibit the activity of elastase as its IC_50_ was very low (0.006 ± 0.004 mg/mL). Moreover, the inhibitory effect on elastase of EGCG was only six times more potent than IF, which was considered to be not significantly different (*p* > 0.05). Therefore, IF was worth using as an elastase inhibitor, whereas, CF was not worth using as an MMP-1 inhibitor since it required a large amount of high concentration of formulation. The present study is the first to reveal anti-elastase of *C. militaris* and *I. tenuipes* extracts. Apart from the extreme effectiveness of IF on elastase inhibition, equalling the effectiveness of EGCG, a natural elastase inhibitor (Andrade et al. 2021), IF was the strongest hyaluronidase inhibitor compared to the others (*p* < 0.05). Therefore, IF was suggested as a potent natural extract, which exerted anti-wrinkle activities.

**Table 5.** Anti-wrinkle activities of *C. militaris* and *I. tenuipes* extracts.

Extracts	IC_50_ (mg/mL)		Hyaluronidase Inhibition (%)
	MMP-1 inhibition	Elastase inhibition	
OA	0.002 ± 0.000^a^	N.D.	81.4 ± 1.6^a^
EGCG	N.D.	0.001 ± 0.000^a^	N.D.
CF	2.00 ± 0.39^b^	1.27 ± 0.01^b^	21.1 ± 1.5^c^
CM	13.29 ± 0.19^d^	5.14 ± 1.53 ^b^	4.6 ± 1.7^d^
IF	5.62 ± 0.54^c^	0.006 ± 0.004^a^	30.3 ± 3.2^b^
IM	25.40 ± 0.36^e^	17.74 ± 4.64^c^	5.5 ± 2.8^d^

IC_50_: a concentration that exhibited 50% inhibitory activity; OA: oleanolic acid; EGCG: epigallocatechin gallate; CF: *C. militaris* fruiting body extract; CM: *C. militaris* mycelium extract; IF: *I. tenuipes* fruiting body extract; IM: *I. tenuipes* mycelium extract; N.D.: not determined. The letter a, b, c, d, and e denote significant differences among samples at *p* < 0.05.

Previous studies on the MMP-1 inhibitory activity of *C. militaris* and *I. tenuipes* extracts indicated cordycepin and adenosine as main components that inhibited the activator protein-1 and suppressed MMP-1 expression (Noh et al. 2009, 2010). Cordycepin has a poor correlation with MMP-1 inhibition, while adenosine has a negligible correlation (Table 6). Phenolic compounds were found to be strongly correlated with the inhibitory activities of not only MMP-1 but also elastase and hyaluronidase.

**Table 6.** The relationship between the chemical constituents and anti-ageing activities of *C. militaris* and *I. tenuipes* extracts.

Chemical constituents	Pearson correlation coefficient (*p*-value)		
	MMP-1 inhibition (IC_50_)	Elastase inhibition (IC_50_)	Hyaluronidase Inhibition (%)
TPC	−0.9521	−0.8414	0.8292
	(0.0479)*	(0.1586)	(0.1708)
TFC	−0.2113 (0.7887)	−0.2712 (0.7288)	0.7613 (0.2387)
Cordycepin	−0.426	−0.3878	−0.246
	(0.574)	(0.6122)	(0.754)
Adenosine	−0.1013	0.08753	0.5585
	(0.8987)	(0.9125)	(0.4415)

Asterisks (*) denote significant strong correlations at *p* < 0.05.

On the other hand, flavonoids have been found to be strongly affected by the hyaluronidase inhibitory activity; due to changes in the microenvironment and hyaluronidase conformation caused by the presence of flavonoids (Smiderle et al. 2014). It has been proposed that placing hyaluronan in topical skincare products or using it as a temporary dermal filling agent could slow down hyaluronan degradation and increase hyaluronan levels in the skin layer, resulting in plumping and youthful skin being achieved (Yusuf et al. 2021). IF, a hyaluronidase inhibitor, is another natural extract that can help to slow the skin ageing process.

### Antityrosinasse activities of *C. militaris* and *I. tenuipes* extracts

**Figure 6.**
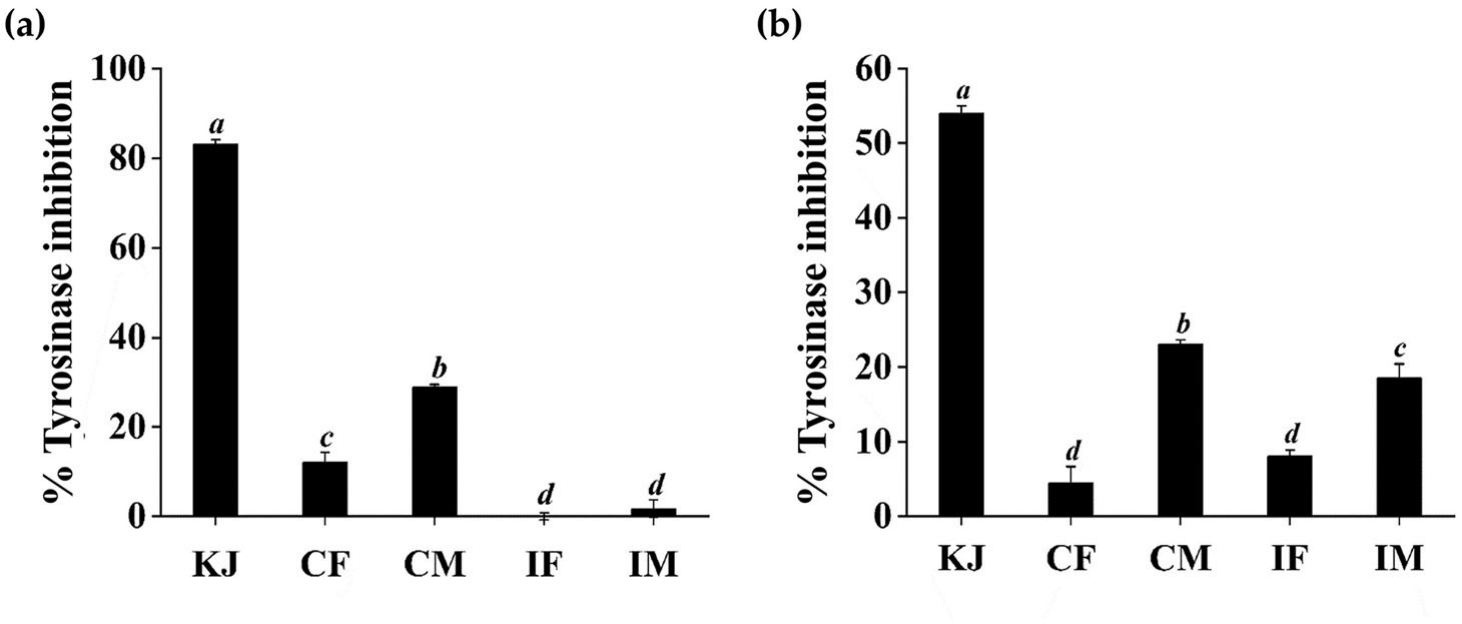
Inhibitory activities against tyrosinase when the substrates were l-tyrosine (a) and L-DOPA (b) of kojic acid (KJ), *C. militaris* fruiting body extract (CF), *C. militaris* mycelium extract (CM), *I. tenuipes* fruiting body extract (IF), and *I. tenuipes* mycelium extract (IM). The letter a, b, c, and d denote significant differences among samples at *p* < 0.05.

Tyrosinase, an enzyme involved in the mechanism of melanogenesis, is found in mammals, plants, and microorganisms. Tyrosinase is also a primary rate-limiting enzyme that catalyses enzyme browning and melanin synthesis. Tyrosinase also exhibits monophenolase and diphenolase activities, which catalyse the hydroxylation of l-tyrosine to L-DOPA and the oxidation of L-DOPA to dopaquinone, which can then be polymerised in a nonenzymatic way to produce dark pigments (Cui et al. 2018). Tyrosinase inhibitors can be used for skin whitening and have been widely used in cosmetics. Kojic acid is a metabolite of fungal that exhibits inhibitory activity towards tyrosinase. The inhibitory effects of kojic acid in this context have been attributed to its ability to chelate copper at the active site of this enzyme. Thus, kojic acid is often used as a positive control to tyrosinase inhibitors. Anti-tyrosinase activities of *C. militaris* and *I. tenuipes* extracts were analysed using l-tyrosine and L-DOPA as a substrate with kojic acid as a reference compound (Figure 6). It was remarked that CM possessed significantly higher tyrosinase inhibitory activity than CF, IF, and IM (*p* < 0.05). The tyrosinase inhibitory activity of CM was 28.9 ± 0.6% and 23.1 ± 0.6% when a substrate was l-tyrosine and L-DOPA, respectively. However, the inhibitory effects of all extracts were less than that with kojic acid.

## Conclusions

Extracts from *C. militaris* and *I. tenuipes* contained a wide range of biologically active compounds, including phenolics, flavonoids, and adenosine, whereas, cordycepin was rarely detected in *I. tenuipes*. Although the chemical profiles of *C. militaris* have previously been reported, the present study focussed on the comparison between different parts, i.e., mycelium and fruiting body of *C. militaris*, as well as the comparison between different fungi, i.e., *C. militaris* and *I. tenuipes*. The findings highlighted that the chemicals varied in the fruiting body and mycelium part. The phenolics and flavonoids in the fruiting body were considerably higher than in the mycelium. Phenolic compounds were found to be moderately correlated with the radical scavenging activities and strongly correlated with the inhibitory activities of MMP-1, elastase, and hyaluronidase. Flavonoids were shown to have a moderate correlation with reducing ability and had a significant impact on hyaluronidase inhibitory function. Adenosine was strongly affected by the lipid peroxidation inhibitory activity (*p* < 0.0006). Apart from the compounds investigated in the present study, other hydrophilic compounds in the extracts may also be responsible for the bioactivities. Therefore, the fractionation of crude extracts to identify key compounds is suggested for further study. IF (*I. tenuipes*) was highlighted as an extract with extraordinary elastase and hyaluronidase inhibitory activities. Furthermore, IF was a strong antioxidant with radical scavenging activity, reducing ability, and lipid peroxidation prevention. Surprisingly, IF had a three-times higher EC_1_ value than ascorbic acid and significantly higher elastase inhibition than EGCG. Therefore, IF is proposed as a powerful natural extract with antioxidant and anti-wrinkle properties.
